# Myeloid Sarcoma Presenting with Leukemoid Reaction in a Child Treated for Acute Lymphoblastic Leukemia

**DOI:** 10.1155/2014/757625

**Published:** 2014-09-03

**Authors:** Aylin Canbolat Ayhan, Cetin Timur, Seyma Sonmez, Ebru Zemheri, Asım Yoruk

**Affiliations:** ^1^Pediatric Hematology-Oncology Department, Goztepe Training Hospital, Istanbul Medeniyet University, 34724 Istanbul, Turkey; ^2^Pathology Department, Goztepe Training Hospital, Istanbul Medeniyet University, 34724 Istanbul, Turkey

## Abstract

*Background.* Myeloid sarcoma is an extramedullary neoplasm of immature myeloid cells. Our study reports a presentation of myeloid sarcoma which presented with severe leukemoid reaction as a secondary malignancy in a patient who was treated for acute lymphoblastic leukemia previously. The case emphasizes the difficulties in diagnosis of patients who do not have concomitant leukemia. *Case Presentation.* A 6-year-old girl who was treated for acute lymphoblastic leukemia previously presented with fatigue, paleness, and hepatosplenomegaly. Peripheral blood smear and bone marrow aspirate examination did not demonstrate any blasts in spite of severe leukemoid reaction with a white cell count 158000/mm^3^. FDG/PET CT revealed slight uptake in cervical and supraclavicular lymph nodes. Excisional lymph node biopsy was performed from these lymph nodes and it showed myeloid sarcoma. *Conclusion.* Myeloid sarcoma can develop as a secondary malignancy in children who are treated for acute lymphoblastic leukemia. It can be associated with severe leukemoid reaction and diagnosis may be difficult if there is not concomitant leukemia. PET/CT is helpful in such cases.

## 1. Introduction

Myeloid sarcoma (MS) is a pathologic diagnosis for an extramedullary proliferation of myeloid lineage blasts which occurs at any site of the body [1]. The most common sites are lymph nodes, skin, bones, and less often the orbits and the central nervous system [2]. It may develop following a previously diagnosed acute myeloid leukemia (AML) or may associate this pathology [1]. MS is reported in 2–14% of patients with AML [3]. In 25% of patients it precedes AML, in 15–35% it appears concomitantly with AML, and in 50% it occurs after the diagnosis of AML [1]. Rarely, MS occurs de novo with no evidence of bone marrow involvement [2]. Our study reports a presentation of myeloid sarcoma with leukemoid reaction as a secondary malignancy in a patient who was treated for acute lymphoblastic leukemia (ALL) previously.

## 2. Case

A six-year-old girl was admitted to hospital with complaints of fatigue and paleness. Previous medical history revealed chemotherapy for common-ALL when she was 2 years old. She was treated at the same center and achieved complete remission. Her maintenance chemotherapy was completed 14 months ago. She was under follow-up monthly, and her last complete blood count (CBC) which was performed one month ago was normal.

On physical examination she was pale. The liver was palpable 2 cm below the right costal margin and the spleen 3 cm below the left costal margin. There was not any enlargement of lymph nodes. Blood tests revealed hemoglobin of 7.6 gr/dL, total white blood cell (WBC) of 28600/mm^3^, and a platelet count of 145000/mm^3^. Peripheral smear examination revealed segmented neutrophils 18%, band neutrophils 13%, lymphocytes 22%, monocytes 27%, promyelocytes 4%, myelocytes 4%, metamyelocytes 10%, eosinophils 1%, and basophil 1%, and there were also normoblasts but this left shift was not accompanied by any blasts. Blood chemistry tests showed an elevated lactate dehydrogenase (LDH) level (708 U/L) and uric acid level (6.6 mg/dL). Renal and liver function tests were normal. We performed bone marrow aspiration for the differential diagnosis and because of her past history especially to see if it was a relapse of ALL or not. Bone marrow aspiration was hypercellular with myeloid hyperactivity and continued differentiation but it did not demonstrate any blasts and was in remission. Flow cytometric immunophenotyping of the bone marrow excluded presence of leukemia. Because of leukemoid reaction and high WBC chronic myeloid leukemia was also suspected but Philadelphia chromosome and BCR/ABL fusion detected in bone marrow aspirate were negative. JAK2 V617F mutation was negative. Cytogenetic study of the bone marrow revealed a normal karyotype. To make differential diagnosis for leukemoid reaction due to a probable infection we evaluated infection markers. C-reactive protein (CRP) was negative. Blood, urine cultures were negative. Urine analysis was normal. Viral serology testings were negative for CMV, Parvovirus, EBV, HIV, Hepatitis A, B and Hepatitis C. On abdominal ultrasound examination there was hepatosplenomegaly. With these laboratory results we excluded myeloid leukemoid reaction due to infection but we were unable to explain the impairment in CBC of the patient. We decided to follow her with weekly repeated CBC and peripheral blood smear for leukocyte differential. WBC continued to increase progressively; leukocyte differential kept to be similar to initial one with presence of promyelocytes, myelocytes, metamyelocytes, granulocytes, and a few normoblasts but no blasts. Liver and spleen enlargement progressed day by day. We performed abdominal MRI but it did not demonstrate any specific finding other than massive hepatosplenomegaly. In the third week WBC was 64000/mm^3^, Hb: 7.2 gr/dL, but this time her PLT count was normal, 259000/mm^3^, and LDH was 654 IU/L. Because of increase in WBC we repeated the bone marrow aspiration and it was very similar to the first one.

Five weeks after the first bone marrow aspiration WBC increased to 158000/mm^3^, Hb was 7.2 gr/dL, and PLT decreased to 64000/mm^3^. At this time the liver was 10 cm, and spleen was 12 cm palpable below the costal margins. We performed bone marrow aspiration once more. It was hypercellular with increased myeloid activity but this time and 4% of the nucleated cells were atypical cells. Flow cytometric immunophenotyping of the bone marrow did not demonstrate any blast population in spite of 4% of blast on smear examination.

**Figure 1:**
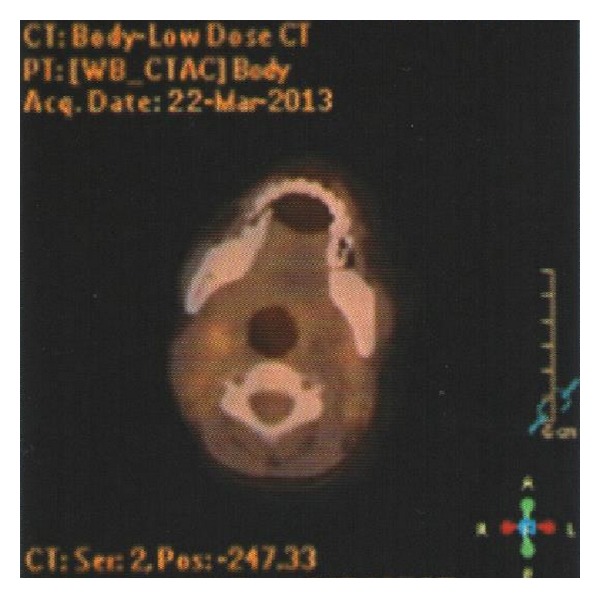
FDG-PET/CT, cervical lymph nodes.

**Figure 2:**
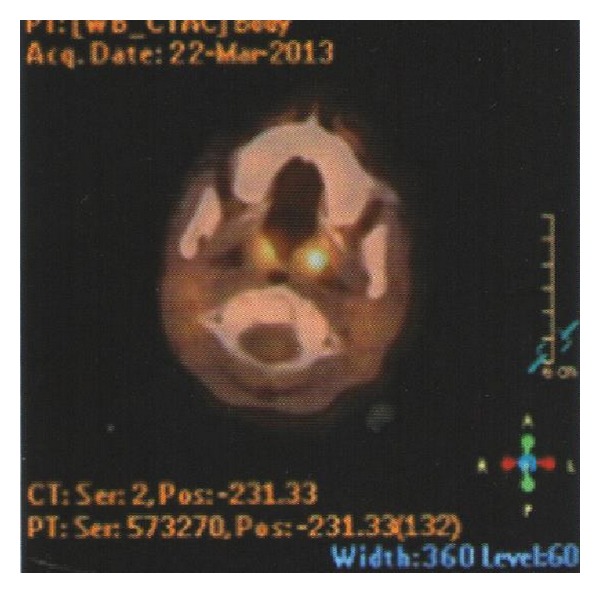
FDG-PET/CT, cervical lymph nodes.

**Figure 3:**
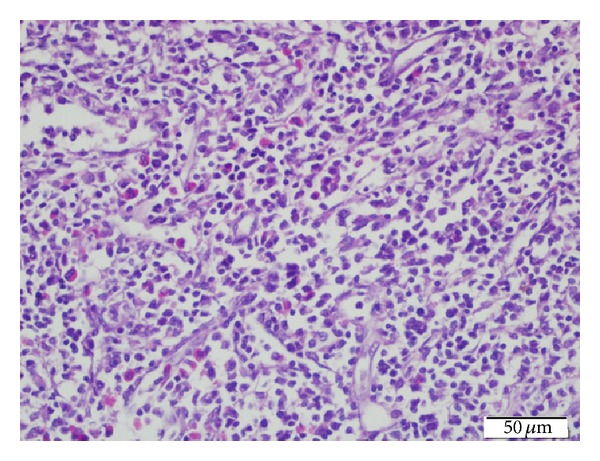
Polymorphic abnormal cells with atypical medium to large sized mononuclear cells admixed with eosinophil precursors (H&E x40).

**Figure 4:**
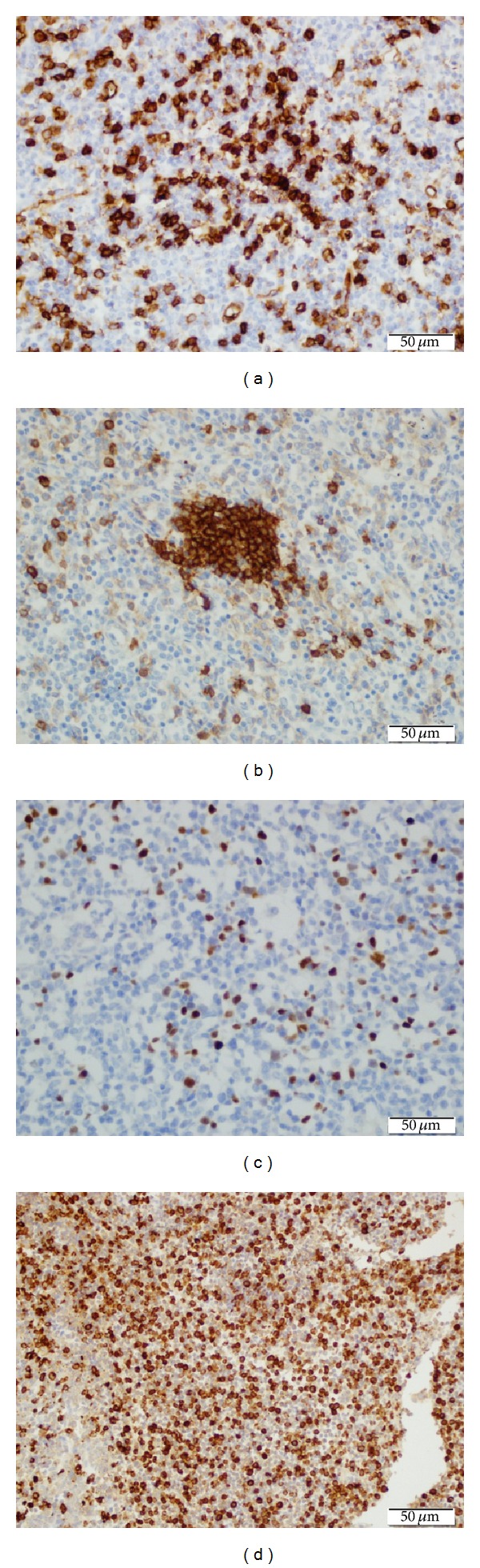
Polymorphic abnormal cells positively stained with A-CD34x40, B-C117x40, C-Tdtx40, and D-MPOx40.

Philadelphia chromosome and BCR/ABL fusion detected in bone marrow aspirate were negative again. JAK2 V617F mutation was repeated too and it was negative. Cytogenetic study of the bone marrow revealed a normal karyotype, and t(15,17), t(8,21), and inv16 were negative. Leukocyte alkaline phosphatase (LAP) score was 16. With all these results we excluded ALL relapse, AML, and chronic myeloid leukemia but we were unable to explain the leukemoid reaction and progressive hepatosplenomegaly. We could not perform spleen aspiration because of the prolonged coagulation profile and severe thrombocytopenia. Because liver function tests remained within normal limits and normal liver parenchyma structure on ultrasonography we did not perform liver biopsy either. A fluorodeoxyglucose (FDG) positron emission tomography (PET) scan was performed and it revealed slight uptake in bilateral cervical and supraclavicular lymph nodes. The largest lymph node was 11 mm, maximal standardized uptake value (SUVmax) of 1.5 (Figures 1 and 2). The proliferation of hypermetabolic lesions was also observed in spleen, bones with SUVmax: 2.7 and SUVmax: 2.1, respectively. We decided to perform lymph nodes biopsy from one of these nodes. In the sixth week of her admission to hospital excisional lymph node biopsy was performed. Histologically, lymph node revealed incomplete effacement of the normal lymph nodal architecture by polymorphic abnormal cells with atypical medium to large sized mononuclear cells with frequent mitoses admixed with eosinophil precursors (Figure 3). Most of the atypical cells show blastic myeloid origin positively stained with CD34, CD117, Tdt, and MPO (Figure 4). Ki-67 was highlighted in 60% of the cells. The diagnosis according to morphological and immunophenotypic features is of a primitive haematopoietic neoplasm with myeloid differentiation.

Prior to chemotherapy peripheral blood smear was evaluated once more and it did not demonstrate any blasts; WBC was 175000/mm^3^. Bone marrow was hypercellular with a blast count of 12% and immunophenotyping demonstrated myeloblasts. Based on these findings she was diagnosed as having myeloid sarcoma with a coexisting AML. She was started on induction chemotherapy for AML. Our patient achieved remission and after chemotherapy she underwent bone marrow transplantation from HLA completely matched brother. She currently remains well.

## 3. Discussion

MS usually presents in the setting of coexisting acute myeloid leukemia, myeloproliferative or myelodysplastic disorders [1, 2]. MS is subclassified into granulocytic, monoblastic, or myelomonocytic MS according to the most abundant cell type [1]. The tumors may occur as extramedullary masses without evidence of leukemia in blood or marrow or in association with AML [3]. MS may occur at diagnosis of AML or may precede it [1]. The presence of MS as an extramedullary disease suggests that there might be an aberrant homing signal for the leukemic blasts precluding the bone marrow localization [1]. Sometimes malignancy can present with leukemoid reaction.

Peripheral white blood cell (WBC) count higher than 50000/mm^3^ with significant increase in early myeloid precursors is called leukemoid reaction [4]. Differential diagnosis of leukemoid reactions should be made with leukemias and other causes such as infections. Neutrophilic leukemoid reactions are usually caused by infections, hemorrhage, drugs, hypersensitivity syndrome, myeloid growth factors, malignancy, and splenectomy [4, 5]. It can also be a paraneoplastic manifestation of a neoplasia [6]. Our case presented with anemia, leucocytosis, and WBC continued to increase up to 158000/mm but in spite of this severe leukemoid reaction we were unable to demonstrate any blasts in peripheral blood smear and bone marrow aspirates.

MS can involve any site of the body but lymph nodes gastrointestinal tract, skin, bones, testes, and soft tissue are the most frequently involved parts [7, 8]. Immunohistochemistry is very valuable in identifying antigens such as CD13, CD33, CD34, CD43, CD45, CD99, CD117, MPO, CD68, and lysozyme which are associated with myeloid lineage. MPO and CD117 are the most sensitive markers for myeloid differentiation in myeloid sarcoma [9, 10]. The definitive diagnosis also requires negative staining for the lymphoid lineages CD3 and CD20 [10]. Trisomy 8, inv (16), t(9,11), t(8,21), 11q23, and del(16q) are the chromosomal abnormalities which are found to be related with MS [2, 11, 12].

JAK2 V617F mutation is found in myeloid tumors and in neoplastic proliferation of the hematopoietic cells of myeloproliferative diseases [13]. Yoshiki et al. reported a case of myeloid sarcoma with JAK2 V617F [13].

It is reported that in the presence of isolated MS the median time to the development of acute leukemia ranges from 5 to 12 months [2]. In our case this period was shorter, almost two months. Previous studies have suggested that patients with isolated MS should receive AML-like chemotherapy [14, 15]. Treatment of isolated MS is not clear yet. Chemotherapy, radiotherapy, bone marrow transplantation, and surgery are the possible therapeutic options for patients with MS. Surgery is used for cases with symptomatic compression due to MS.

Delays in treatment of isolated MS almost always result in progression to AML [2, 11]. FDG-PET is sensitive for the detection of malignant tumors with increased metabolism. In previous studies it is reported that FDG-PET is effective at detecting or management of MS and might be used to monitor patients with MS [7, 16, 17]. In our case FDG-PET/CT aided us in deciding the biopsy. On physical examination her palpable lymph nodes were smaller than 1 cm and they did not show progress by time because of this at first we did not decide to perform excisional biopsy to any of these lymph nodes. PET/CT was very helpful in this way.

In conclusion diagnosis ofmyeloid sarcoma may be difficult in spite of severe leukemoid reaction and hepatosplenomegaly if there is not concomitant leukemia. FDG-PET/CT is useful in diagnosis of such difficult cases.
